# Supplementary material for: Serine peptidase Vpr forms enzymatically active fibrils outside Bacillus bacteria revealed by cryo-EM
Source: Nat Commun. 2023 Nov 18;14:7503. doi: 10.1038/s41467-023-43359-z (PMC10657474; doi:10.1038/s41467-023-43359-z)
Supplement: Supplementary file 1 — Supplementary Information [file 41467_2023_43359_MOESM1_ESM.pdf]

Title: Serine peptidase Vpr forms enzymatically active fibrils outside *Bacillus* bacteria revealed by cryo-EM

Authors: Yijia Cheng[1], Jianting Han[1], Meinai Song[1], Shuqin Zhang[1], Qin Cao[1*]

Affiliations:
[1]Bio-X Institutes, Key Laboratory for the Genetics of Developmental and Neuropsychiatric Disorders, Ministry of Education, Shanghai Jiao Tong University, Shanghai, 200030, China
[*]Correspondence to: Qin Cao, email: caoqin@sjtu.edu.cn

Inventory of Supporting Information

Supplementary Figures 1-6

Supplementary Tables 1 and 2

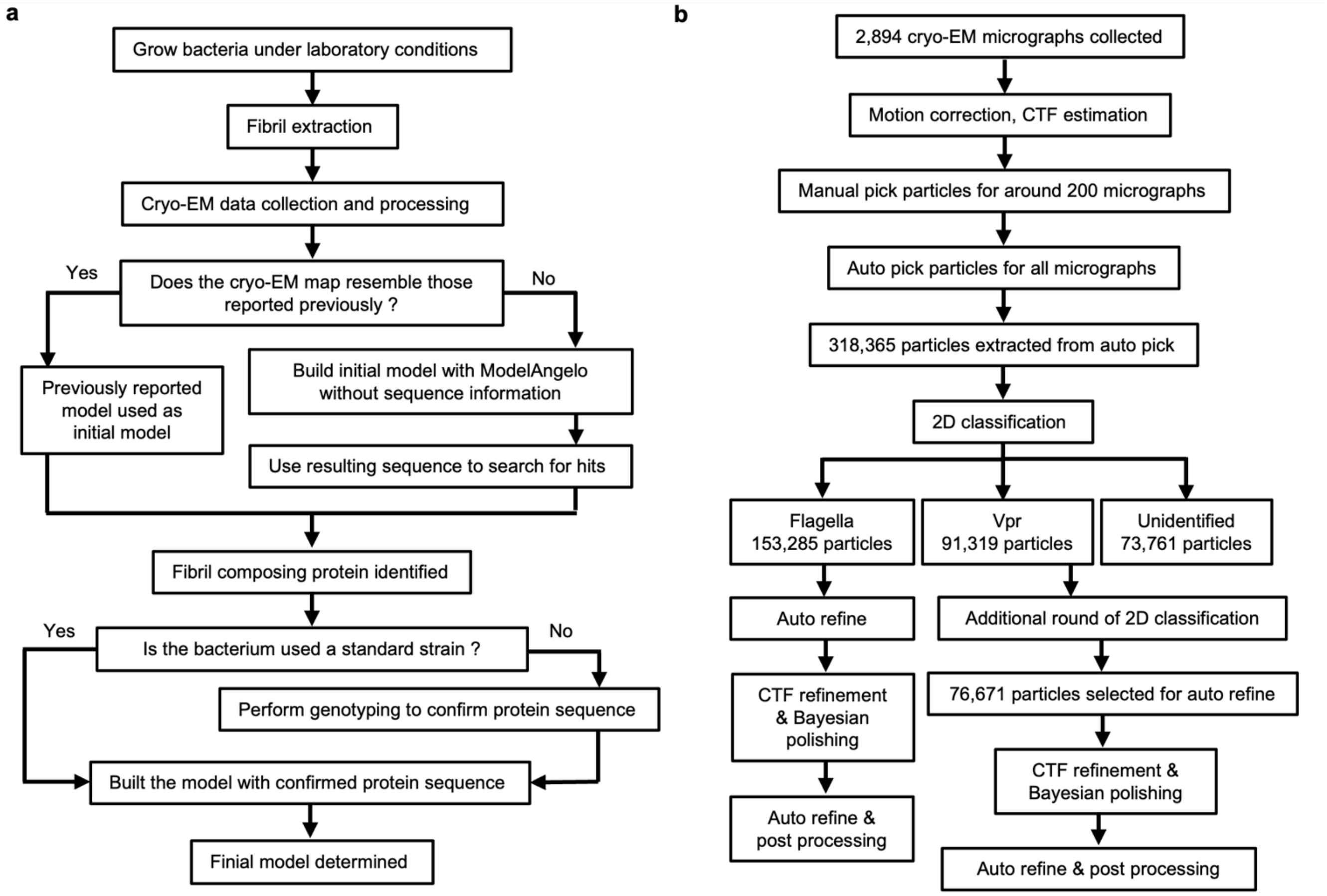

Supplementary Figure 1 Workflow of fibril extraction and cryo-EM structure determination. (**a**) Overall strategy for fibril structure determination from bacterial biofilm. (**b**) Workflow of cryo-EM data processing.

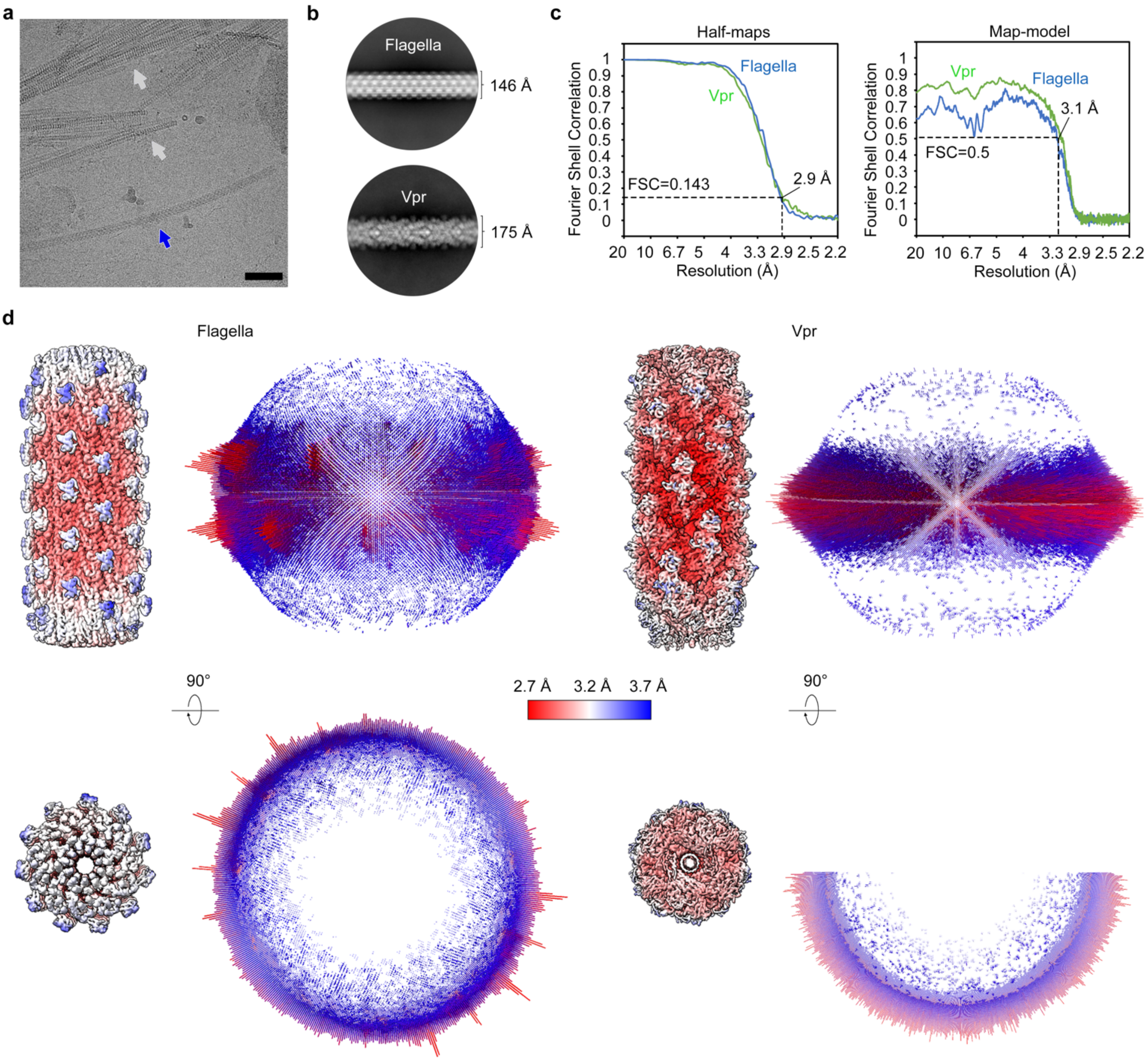

Supplementary Figure 2 Cryo-EM data processing. (**a**) Representative cryo-EM micrograph of *Bacillus amyloiquefaciens* fibrils, indicating representative fibrils categorized as flagella (blue) and unidentified (grey) with arrows. The scale bar is 100 nm. A total of 2,894 micrographs has been collected in this study, and flagella (blue) and unidentified (grey) fibrils can be observed in most of these micrographs. (**b**) Two-dimensional (2D) classes of two fibril species identified in this study. (**c**) FSC curves between two half-maps (left panel) and the cryo-EM reconstruction and refined atomic model (right panel). (**d**) Local resolution estimate (left) and angular distribution of particles used in the final reconstruction (right) of flagella and Vpr fibrils. Local resolution maps are colored from red (2.7 Å) to blue (3.7 Å).

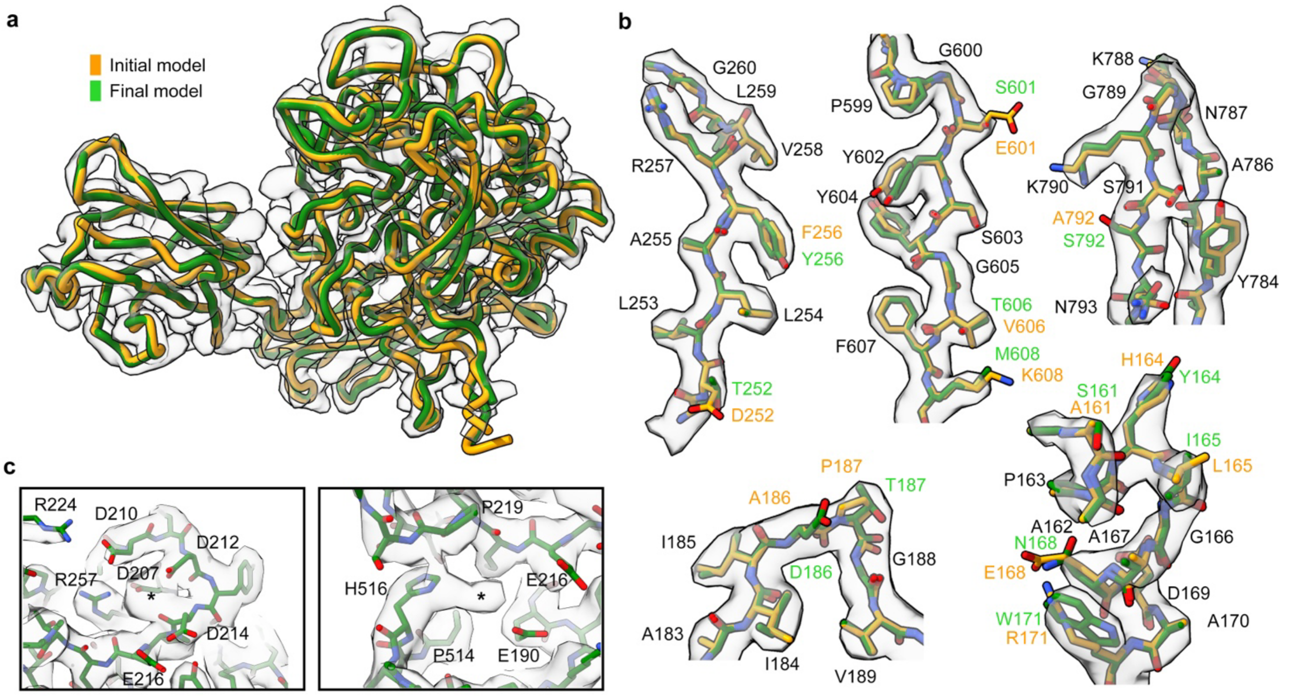

Supplementary Figure 3: Atomic model building of Vpr fibrils. (**a**) Cryo-EM map and superimposition of the initial model (generated by ModelAngelo) and final model of Vpr. (**b**) Detailed comparison of the initial model and final model of Vpr. We note that both the initial and final models fit the cryo-EM map well. (**c**) Extra densities adjacent to Asp212 (left panel) and His516 (right panel) in the Vpr map, which may suggest potential ligand bindings or post-translational modifications. Extra densities are indicated with asterisks (*).

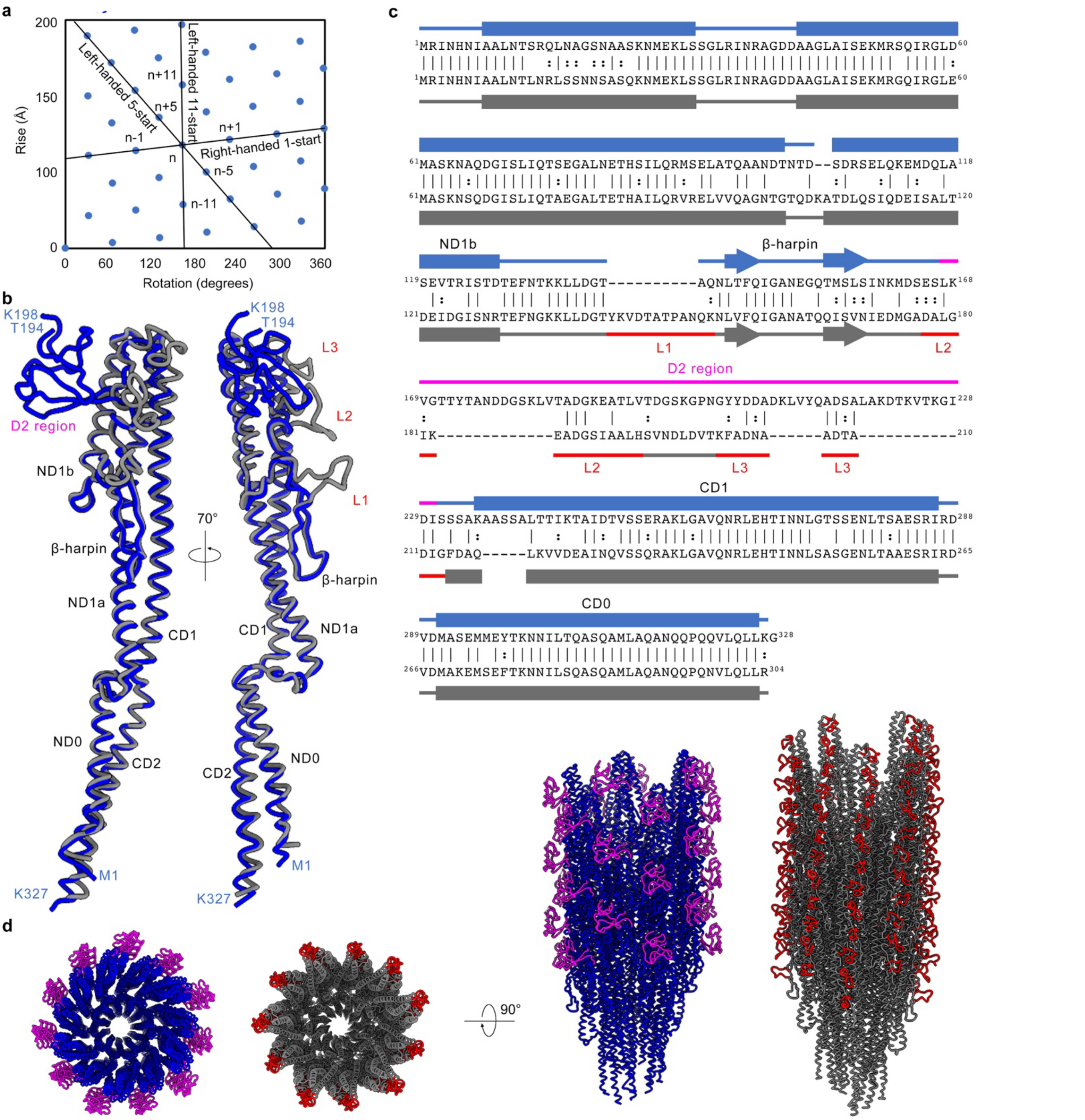

Supplementary Figure 4 Structural analysis of flagellar fibrils. (**a**) The helical net of the *Bacillus amyloiquefaciens* flagellar fibrils. (**b**) Superimposition of *Bacillus amyloiquefaciens* (blue, this study) and *Bacillus subtilis* (grey, PDB ID 5WJT) flagellin structures. (**c**) Sequence alignment of *Bacillus amyloiquefaciens* (top) and *Bacillus subtilis* (bottom) flagellin. Secondary structures of *Bacillus amyloiquefaciens* and *Bacillus subtilis* flagellin were aligned with their amino acid sequence and colored in blue and grey, respectively. The residue range of the extra loops in *Bacillus subtilis* flagellin were colored in red, and that of the D2 region in *Bacillus amyloiquefaciens* flagellin were colored in magenta. (**d**) Top views (left panels) and side views (right panels) of *Bacillus amyloiquefaciens* and *Bacillus subtilis* flagellar fibril structures. The extra loops (L1, L2, and L3) in *Bacillus subtilis* flagellin were colored in red, and the D2 domains in *Bacillus amyloiquefaciens* flagellin were colored in magenta.

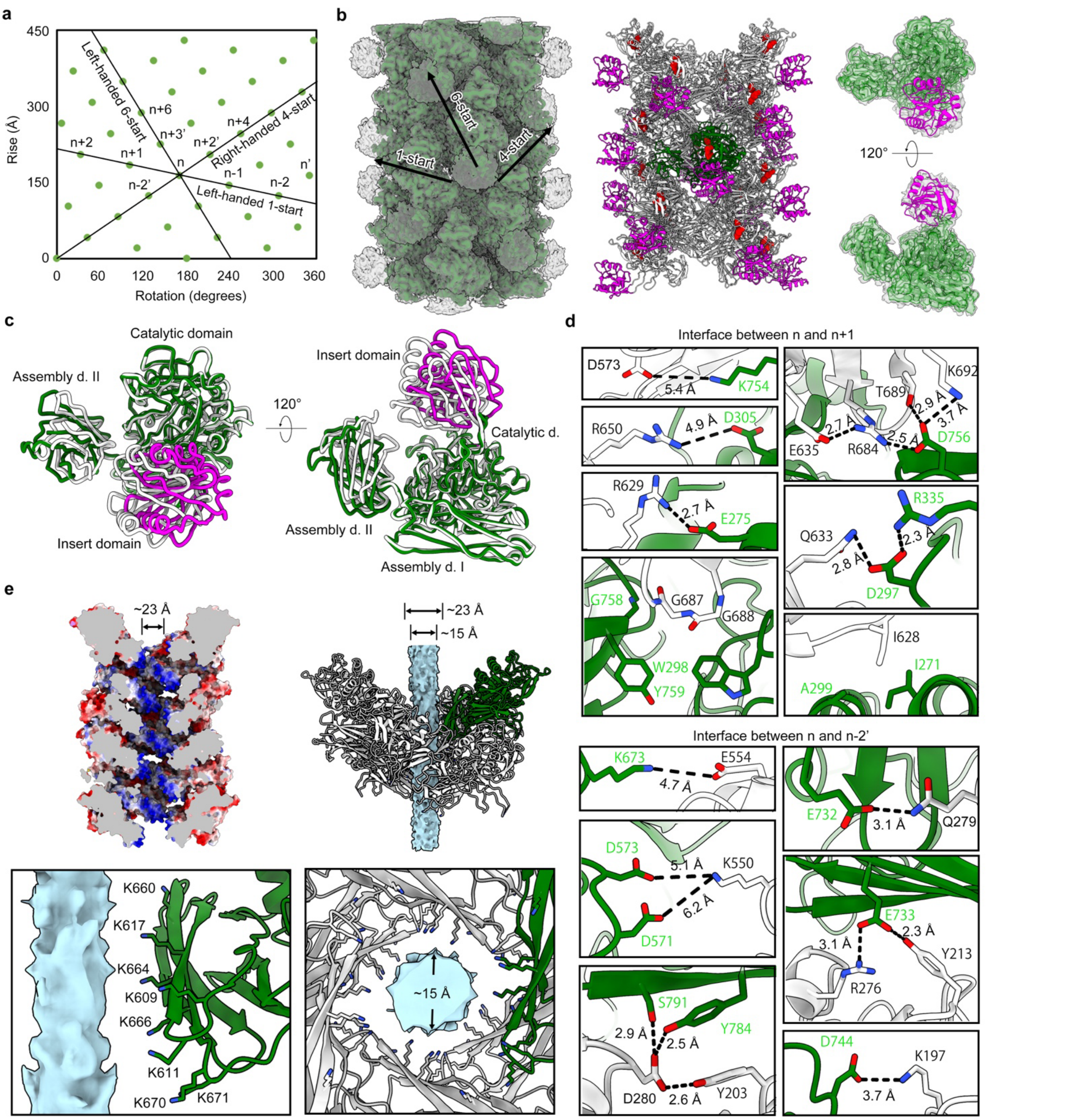

Supplementary Figure 5 Structural analysis of Vpr fibrils. (**a**) The helical net of Vpr fibrils. Subunit n and n' are related by C_2 symmetry. (**b**) The position and overall shape of the insert domain in Vpr fibrils. The left panel shows the cryo-EM map of Vpr fibrils in high (green) and low (grey) thresholds. The maps of the insert domain is only visible in the low threshold. The middle panel shows the atomic model of Vpr fibrils, with one subunit colored in green, active sites of all subunits colored in red, and insert domains colored in magenta. The insert domains were modeled by docking the alpha-fold model into the low threshold map as a rigid body. The right panel shows the cryo-EM map (high threshold in green and low threshold in grey) and model of one subunit. (**c**) Superimposition of the cryo-EM model (green and magenta) and alpha-fold model (white) of Vpr. The overall fold of the two models is very similar, and the relative positions of the assembly domain II and the insert domain were slightly shifted. (**d**) The details of interactions between adjacent subunits in Vpr fibrils. The hydrogen bonds and salt bridges were indicated by dashed lines. (**e**) The extra densities inside Vpr fibrils. The top-left panel shows the electrostatic diagrams showing the inner surface of Vpr fibrils. The top-right panel shows a side view of six Vpr subunits (one in green and the others in white) surrounding the extra densities (shown in light blue, the threshold of the density was the same as the high

threshold in panel b). The bottom-left panel shows side views of one subunit and the extra densities, with the side chains of lysine residues adjacent to the extra densities shown in sticks. The bottom-right panel shows a top view of six Vpr subunits and the extra densities.

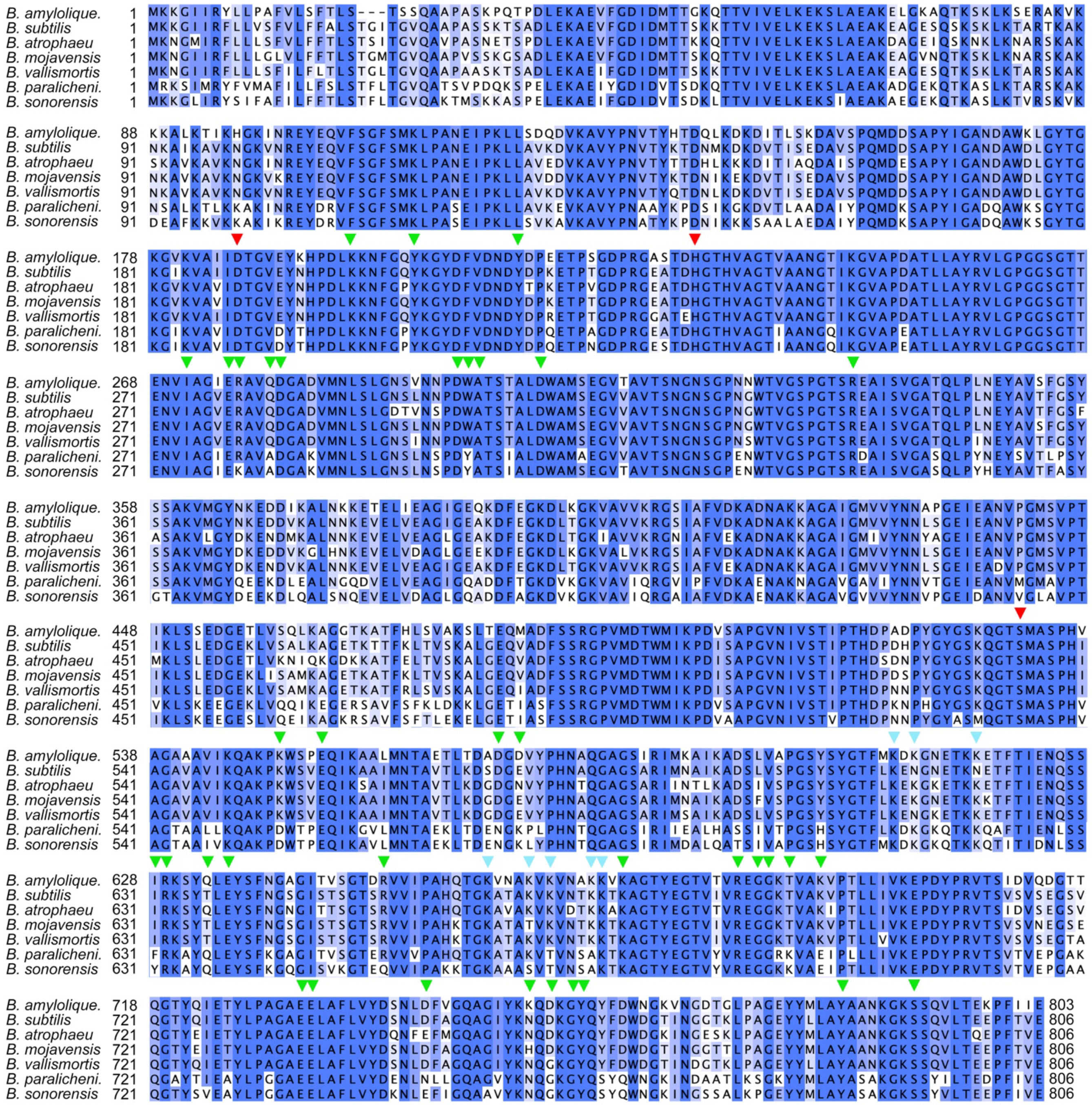

Supplementary Figure 6: Sequence Alignment of Vpr Protease in representative *Bacillus* bacteria. Residues composing the catalytic triad were indicated with red arrows. Residues involved in inter-subunit interactions are indicated with green arrows. Lysine residues surrounding the extra densities in the inner channel of Vpr fibrils were indicated with blue arrows. Note that most of residues involved in inter-subunit interactions are conservative or replaced with similar residues. The exceptions are as follows: i) in *B. paralicheni*, *B. sonorensis*, and *B. vallismortis*, Asp756 was replaced by a glycine residue, which diminishes the interaction network between Asp756, Arg684, Thr689, and Lys692; ii) in *B. sonorensis*, *B. mojavensis*, and *B. vallismortis*, Gln633 was replaced by threonine, which should diminish the hydrogen bond between Gln633 and Asp297; iii) in *B. paralicheniformis* and *B. sonorensis*, Gln279 was replaced by alanine, which should diminish the hydrogen bond between Gln279 and Glu732; iv) a long-distance salt bridge between Lys754 and Asp573 exists only in operational group *B. amyloliquefaciens* because of sequence variation. We believe that all these exceptions have limited influences on subunit interaction in Vpr fibrils, so that Vpr should also be able to assemble into fibrils in these bacteria.

Supplementary Table 1 Primer sequences

Primers	Sequences (from 5' to 3')
Universal primers 27F	AGAGTTTGATCCTGGCTCAG
Universal primers 1492R	TACGGTTACCTTGTTACGACTT
Hag-forward	TCCTGGAAGAGAACAACAGAGC
Hag-reverse	TTGGCTTGCTGAAGGAGGAG
Vpr-forward	AACTCCCTCCTTATTCACAGAC
Vpr-reverse	ATACCGTCTGCTTCGAGAAG

Supplementary Table 2 Genotyping results

Genotyping	Sequences (from 5' to 3')
16S rRNA	CGGCTGGCTCCATAAAGGTTACCTCACCGACTTCGGGTGTTACAAACTCTCGTGGTGTGA<br>CGGGCGGTGTGTACAAGGCCCGGGAACGTATTCACCGCGGCATGCTGATCCGCGATTAC<br>TAGCGATTCCAGCTTCACGCAGTCGAGTTGCAGACTGCGATCCGAACTGAGAACAGATT<br>TGTGGGATTGGCTTAACCTCGCGGTTTCGCTGCCCTTTGTTCTGTCCATTGTAGCACGTGT<br>GTAGCCCAGGTCATAAGGGGCATGATGATTTGACGTCATCCCCACCTTCCTCCGGTTTGT<br>CACCGGCAGTCACCTTAGAGTGCCCAACTGAATGCTGGCAACTAAGATCAAGGGTTGCG<br>CTCGTTGCGGGACTTAACCCAACATCTCACGACACGAGCTGACGACAACCATGCACCAC<br>CTGTCACTCTGCCCCCGAAGGGGACGTCCTATCTCTAGGATTGTCAGAGGATGTCAAGA<br>CCTGGTAAGGTTCTTCGCGTTGCTTCGAATTAAACCACATGCTCCACCGCTTGTGCGGGC<br>CCCCGTCAATTCCTTTGAGTTTCAGTCTTGCGACCGTACTCCCCAGGCGGAGTGCTTAAT<br>GCGTTAGCTGCAGCACTAAGGGGCGGAAACCCCCTAACACTTAGCACTCATCGTTTACG<br>GCGTGGACTACCAGGGTATCTAATCCTGTTCGCTCCCCACGCTTTCGCTCCTCAGCGTCA<br>GTTACAGACCAGAGAGTCGCCTTCGCCACTGGTGTTCCTCCACATCTCTACGCATTTCAC<br>CGCTACACGTGGAATTCCACTCTCCTCTTCTGCACTCAAGTTCCCCAGTTTCCAATGACC<br>CTCCCCGGTTGAGCCGGGGGCTTTCACATCAGACTTAAGAAACCGCCTGCGAGCCCTTT<br>ACGCCCAATAATTCCGGACAACGCTTGCCACCTACGTATTACCGCGGCTGCTGGCACGT<br>AGTTAGCCGTGGCTTTCTGGTTAGGTACCGTCAAGGTGCCGCCCTATTTGAACGGCACTT<br>GTTCTTCCCTAACAACAGAGCTTTACGATCCGAAAACCTTCATCACTCACGCGGCGTTGC<br>TCCGTCAGACTTTCGTCCATTGCGGAAGATTCCCTACTGCTGCCTCCCGTAGGAGTCTGG<br>GCCGTGTCTCAGTCCCAGTGTGGCCGATCACCCTCTCAGGTCGGCTACGCATCGTCGCCT<br>TGGTGAGCCGTTACCTCACCAACTAGCTAATGCGCCGCGGGTCCATCTGTAAGTGGTAG<br>CCGAAGCCACCTTTTATGTCTGAACCATGCGGTTCAGACAACCATCCGGTATTAGCCCCG<br>GTTTCCCGGAGTTATCCCAGTCTTACAGGCAGGTTACCCACGTGTTACTCACCCGTCCGC<br>CGCTAACATCAGGGAGCAAGCTCCCATCTGTCCGCTCGACTTGCA
Flagellin	ATGAGAATCAACCACAATATCGCGGCTCTTAACACTAGCCGTCAGCTGAATGCAGGTTC<br>AAACGCTGCTTCTAAAAACATGGAAAAATTATCTTCAGGTCTTCGCATCAACCGCGCTG<br>GTGATGACGCTGCGGGTCTTGCGATCTCTGAAAAAATGCGTTCTCAAATCCGCGGTTTAG<br>ACATGGCGTCTAAAAACGCTCAAGACGGAATCTCTCTTATCCAAACATCTGAGGGTGCA<br>TTGAACGAAACTCACAGCATTCTTCAGCGTATGAGCGAGCTTGCTACACAAGCGGCAAA<br>CGATACAAACACAGATTCTGACCGTTCTGAGCTTCAAAAAGAGATGGACCAATTAGCGT<br>CTGAAGTAACAAGAATCTCTACTGACACTGAGTTCAACACGAAGAAACTTCTTGACGGA<br>ACTGCGCAAAACCTGACGTTCCAAATCGGAGCTAACGAAGGCCAAACAATGAGCCTGTC<br>TATCAATAAAATGGACTCTGAAAGCCTGAAAGTTGGTACGACTTACACAGCAAATGATG<br>ACGGTTCTAAGCTTGTAACTGCAGATGGAAAAGAAGCAACACTTGTCACTGACGGTTCT<br>AAAGGGCCGAATGGCTACTATGACGATGCTGATAAATTAGTTTATCAAGCTGACAGTGC<br>ATTAGCTAAAGATACAAAAGTAACAAAAGGTATCGACATCTCTTCTTCAGCTAAAGCTG<br>CGTCTTCAGCTCTTACAACAATCAAAACAGCTATCGACACAGTATCTAGCGAGCGCGCT

	AAACTTGGTGCGGTTCAAAACCGTTTAGAGCACACAATCAACAACCTTGGTACTTCTTCT<br>GAGAACCTGACTTCTGCTGAATCACGTATCCGTGACGTAGACATGGCTTCTGAGATGAT<br>GGAGTACACGAAAAACAACATCCTTACTCAGGCTTCTCAAGCTATGCTTGCGCAAGCTA<br>ACCAACAGCCTCAGCAAGTTCTTCAATTGCTTAAAGGTTAA
Vpr	TTGAAAAAAGGAATCATCCGTTATCTGCTTCCGGCTTTTGTCTTATCCTTTACCTTATCCA<br>CAAGTTCACAGGCTGCGCCGGCTTCAAAACCGCAAACTCCTGATCTTGAGAAGGCTGAG<br>GTATTTGGTGACATTGATATGACCACCGGCAAACAAACGACAGTCATCGTCGAGCTGAA<br>GGAAAAGTCACTGGCGGAAGCAAAGGAACTCGGCAAAGCACAGACAAAAAGCAAGCT<br>GAAAAGCGAACGCTCAAAAGTGAAAAAGAAAGCGCTCAAAACCATTAAACACGGAAAA<br>ATCAACAGGGAATATGAGCAAGTGTTTTCCGGCTTCTCCATGAAACTCCCGGCAAATGA<br>AATTCCGAAACTGCTGAGTGATCAGGATGTCAAAGCGGTTTATCCGAACGTCACCTATC<br>ATACCGATCAGCTGAAAGATAAAGACATCACCCTATCTAAGGATGCCGTGTCTCCGCAG<br>ATGGATGACAGCGCGCCTTATATAGGGGCAAATGACGCGTGGAAGCTCGGCTATACGGG<br>GAAAGGCGTCAAGGTGGCCATTATTGACACCGGTGTCGAATATAAACACCCTGACTTAA<br>AGAAAAATTTCGGACAATATAAAGGATACGATTTTGTGGATAACGATTACGATCCTGAA<br>GAAACGCCGTCCGGTGATCCGAGAGGCGCGTCGACTGACCACGGAACCCATGTCGCGG<br>GCACGGTAGCGGCAAACGGAACGATTAAAGGCGTAGCGCCGGATGCTACACTCCTTGCC<br>TACCGTGTGCTCGGTCCGGGCGGAAGCGGAACAACGGAGAACGTCATCGCCGGTATTGA<br>ACGCGCCGTACAGGACGGAGCGGATGTCATGAACCTTTCTCTCGGCAATTCTGTGAATA<br>ACCCAGACTGGGCGACAAGCACGGCGCTTGACTGGGCGATGTCAGAAGGCGTCACGGC<br>CGTTACATCAAACGGAAACAGCGGGCCGAACAATTGGACCGTCGGCTCTCCGGGAACGT<br>CCAGAGAAGCTATCTCCGTCGGAGCGACACAGCTGCCGCTGAACGAGTACGCCGTCTCC<br>TTCGGTTCCTATTCCTCAGCGAAAGTAATGGGCTACAACAAAGAAGATGACATAAAAGC<br>ACTGAATAAAAAAGAAACAGAGCTCATAGAGGCGGGTATCGGCGAGCAAAAGGATTTT<br>GAAGGCAAAGATCTGAAAGGAAAAGTCGCGGTCGTCAAACGGGGCAGCATCGCCTTTG<br>TGGATAAAGCAGACAACGCCAAAAAAGCGGGCGCGATCGGTATGGTTGTGTATAACAA<br>TGCCCCGGGAGAAATTGAAGCCAACGTACCGGGCATGTCCGTGCCGACGGTTAAGCTTT<br>CATCAGAAGACGGCGAAAAACTCGTCAGCCAATTAAAAGCGGGCGGCACAAAAGCGAC<br>ATTCCATTTATCCGTGGCTAAATCGCTCACTGAACAAATGGCAGACTTTTCGTCACGCGG<br>TCCGGTTATGGACACGTGGATGATTAAACCTGACGTCTCCGCTCCCGGCGTAAACATCGT<br>CAGCACCATTCCGACCCATGATCCGGCCGACCCGTACGGCTACGGATCGAAGCAGGGAA<br>CGAGCATGGCTTCCCCGCATGTAGCCGGCGCAGCTGCCGTCATCAAGCAGGCCAAACCG<br>AAATGGAGCCCGGAACAAATAAAAGCCGCTCTCATGAACACGGCGGAAACCTTAACGG<br>ACGCGGACGGTGACGTATACCCGCATAATGCGCAAGGCGCCGGAAGCATCAGAATCAT<br>GAAGGCGATCAAAGCAGACTCCCTTGTCGCCCCGGGAAGTTATTCTTACGGAACGTTTA<br>TGAAAGACAAAGGCAATGAAACGAAAAAAGAAACCTTTACGATTGAAAACCAATCGTC<br>CATCAGAAAATCATATCAGCTCGAGTACTCTTTCAACGGCACGGGCATTACCGTTTCGG<br>GCACAGACCGGGTCGTGATCCCCGCTCATCAAACCGGAAAGGTCAATGCGAAAGTAAA<br>GGTCAATGCCAAAAAAGTAAAGGCAGGCACCTATGAGGGAACCGTCACAGTGCGTGAA<br>GGCGGAAAAACAGTCGCAAAAGTGCCGACGCTGCTGATTGTAAAAGAGCCGGACTATC<br>CGCGCGTAACGTCAATCGACGTACAGGACGGCACAACGCAGGGAACCTACCAAATTGA<br>AACCTATCTTCCGGCTGGAGCCGAAGAGCTTGCCTTCCTTGTATATGACAGTAATCTTGA<br>CTTTGTCGGCCAAGCCGGCATCTACAAAAAGCAGGATAAAGGCTATCAATATTTCGACT<br>GGAACGGCAAAGTCAATGGTGACACCGCACTGCCGGCAGGAGAATATTATATGCTGGCC<br>TATGCCGCGAACAAAGGGAAATCAAGCCAGGTGCTGACTGAAAAACCTTTTATCATTGA<br>ATAA
